# The Burden of Acute Disease in Mahajanga, Madagascar – A 21 Month Study

**DOI:** 10.1371/journal.pone.0119029

**Published:** 2015-03-04

**Authors:** Vijay C. Kannan, Clara N. Andriamalala, Teri A. Reynolds

**Affiliations:** 1 University of Texas Southwestern, Division of Emergency Medicine, Dallas, Texas, United States of America; 2 Department of Emergency Medicine, Maricopa Medical Center, Phoenix, Arizona, United States of America; 3 Centre Hôpitalier Universitaire de Mahajanga, Mahajanga, Madagascar; 4 Muhimbili National Hospital, Emergency Medicine Department, Dar Es Salaam, Tanzania; 5 University of California San Francisco, Emergency Medicine and Global Health Sciences, San Francisco, California, United States of America; University of Florida College of Medicine, UNITED STATES

## Abstract

**Background:**

Efforts to develop effective and regionally-appropriate emergency care systems in sub-Saharan Africa are hindered by a lack of data on both the burden of disease in the region and on the state of existing care delivery mechanisms. This study describes the burden of acute disease presenting to an emergency unit in Mahajanga, Madagascar.

**Methods and Findings:**

Handwritten patient registries on all emergency department patients presenting between 1 January 2011 and 30 September 2012 were reviewed and data entered into a database. Data included age, sex, diagnosis, and disposition. We classified diagnoses into Clinical Classifications Software (CCS) multi-level categories. The population was 53.5% male, with a median age of 31 years. The five most common presenting conditions were 1) Superficial injury; contusion, 2) Open wounds of head; neck; and trunk, 3) Open wounds of extremities, 4) Intracranial injury, and 5) Unspecified injury and poisoning. Trauma accounted for 48%, Infectious Disease for 15%, Mental Health 6.1%, Noncommunicable 29%, and Neoplasms 1.2%. The acuity seen was high, with an admission rate of 43%. Trauma was the most common reason for admission, representing 19% of admitted patients.

**Conclusions:**

This study describes the burden of acute disease at a large referral center in northern Madagascar. The Centre Hôpitalier Universitaire de Mahajanga sees a high volume of acutely ill and injured patients. Similar to other reports from the region, trauma is the most common pathology observed, though infectious disease was responsible for the majority of adult mortality. Typhoid fever other intestinal infections were the most lethal CCS-coded pathologies. By utilizing a widely understood classification system, we are able to highlight contrasts between Mahajanga’s acute and overall disease burden as well as make comparisons between this region and the rest of the globe. We hope this study will serve to guide the development of context-appropriate emergency medicine systems in the region.

## Background

The Disease Control Priorities project estimates that 45% of deaths and 36% of Disability-Adjusted Life Years (DALYs) could be mitigated by effective emergency care systems, and World Health Assembly Resolution 60.22 has called for the development of such systems in all of its member states.[1]. However, efforts to develop effective and regionally-appropriate systems in sub-Saharan Africa have been hindered by a lack of data on both the burden of acute disease in the region and on the state of existing care delivery mechanisms [2]. The burden of acute disease, or rather the incidence of the various pathologies seen at an emergency care facility, suffers from a lack of standardized reporting methodology in current literature. By reporting this using an international classification system, the potential for meaningful comparisons arises.[3–5] This study aims to describe the burden of disease presenting to an emergency unit in Mahajanga, Madagascar using an ICD-9 based classification system. More granular documentation of the local burden of acute disease may serve to facilitate the development of targeted emergency care delivery systems.[6,7]

The Republic of Madagascar is the fourth largest island in the world, located roughly 200 miles off the eastern coast of Mozambique. It covers an area of nearly 600,000 square kilometers, and is home to a population of 20 million, 70% of who live in rural areas. The 2011 gross national income per capita was $950, classifying it as a low-income country. The median age is 18 years, and life expectancy at birth in 2010 was 63 years for males and 67 years for females. Infant mortality was reported at 76 per 1000 births in 2004, compared with an average of 100 per 1000 in the AFRO region. The World Health Organization (WHO) reports the top five causes of death in the Malagasy people as Lower Respiratory Infections (14%), Malaria (11%), Diarrheal Diseases (9%), Perinatal Conditions (7%), and Measles (5%) [8]. HIV prevalence amongst adults aged 15 to 49 years was reported at 1.7 in 2003, compared to 7.1 in the AFRO region. [9]

The Centre Hôpitalier de Mahajanga is a 400-bed hospital and the only referral center in the Boeny region. It is one of two teaching hospitals in the country and is located in Mahajana, a regional capital with a population of 135,000. While there are no published data regarding admission rates from this region, the catchment area is estimated at 543,000 based on the last population survey of the Boeny region in 2004. Our study took place in the Unité des Urgences et Soins Intensifs, a mixed function unit that serves both as a primary acute care intake area and as an inpatient unit for critically ill patients. This unit does not see pregnant or pediatric patients, these are primarily handled at the adjacent Centre Mère-Enfant. The unit is split into two wings, a medical and a surgical wing. The data from this study was collected from both wings. Each wing is staffed by an on-call attending, a resident, a senior medical student, and a junior medical student. Because emergency medicine is not recognized or taught as an independent specialty in Madagascar, ED house staff is comprised of internists in the medical wing and general surgeons in the surgical wing, along with their respective residents. Medical students complete an 8 to 12 week rotation covering both wings. Patients may present via self-referral, police referral, or referral from any primary or secondary clinic. [10]

## Methods

This study was reviewed and approved by the Institutional Review Committee of Research at CHU Mahajanga, and exempted from review by the Committee on Human Research at UCSF. Data was anonymized and de-identified upon entry. Handwritten patient registries on all emergency department patients presenting between 1 January 2011 and 30 September 2012 were reviewed and data entered by the department chief of service into a Microsoft Excel spreadsheet. Data collected included age, sex, date of service and discharge, diagnoses, and disposition.

### Exclusion Criteria

All patients for whom complete registry data was available were included in analysis. Patients who had no diagnosis listed, and those who were declared “dead on arrival” and not evaluated or treated in the unit, were excluded from analysis.

### Data Analysis

We manually classified diagnoses into Clinical Classifications Software (CCS) multi-level categories. All CCS categorizations were reviewed by at least two physician researchers, and a third physician researcher mediated any disagreements. Aggregate disease categories (trauma, infectious, noncommunicable, etc.) were formed from groups of these codes. For example, the infectious disease aggregate category is comprised of the entirety of CCS code 1 (infectious and parasitic diseases), as well as select samples found in other codes such as encephalitis (6.1.2, found in neurologic diseases), and pneumococcal pneumonia (8.1.1.1, found in respiratory). We created these aggregate categories to offer an additional way of viewing the data. These are categories commonly used in order to understand the relative contribution of key groups of conditions to the overall emergency care burden. They reflect groups of conditions identified as high priorities by the WHO and which tend to align with distinct funding pathways.[9,11,12]

## Results

There were 5,154 patients seen in the department during the study period. Registry data was complete for 5,138 patients (99.7%) and these were included in the analysis. Sixteen patients (0.3%) had incomplete information and were excluded from analysis.

Table 1 displays the demographic breakdown of our study population. We observed a 53.5% male predominance, with a median age (of all sexes) of 31 years. The median age of men (34 years) was slightly higher than that of women. Gender data was unavailable for 59.8% of pediatric and adolescent cases (<18yrs), as gender is not commonly entered for children under 15 at this site. This is due to the fact that pediatric and pregnant cases are traditionally handled at the adjacent Centre Mère-Enfant, not investigated during this study.

**Table 1:** Patient Demographics.

	ALL	M	F	Unknown Sex
Median age	31	34	31	7*
Age categories				
<5	138 (2.7%)	1	2	135
<18	844 (16.4%)	144	195	505
≥18	4263 (83.0%)	2585	1678	0
Missing age	31 (0.6%)	22	9	0
TOTAL	5138	2751	1882	505

* Gender information is commonly not entered for children under 15 at this site, resulting in the low median age in this category.

Table 2 depicts select aggregate disease categories frequently investigated by epidemiologic studies. Though trauma is the most common disease category at CHUM overall, its contribution to mortality varies by age. Trauma accounts for only 9.8% of deaths overall and 8% of deaths in adults, but 31% of deaths in children.

**Table 2:** Select Aggregate Disease Categories by Age Group and by Mortality.

	Disease Category	All Ages (% of total)	<5 (% of total)	<18 (% of total)	≥18 (% of total)
**All Patients**	**Trauma**	47.94	82.61	69.55	43.65
	**Infectious**	14.67	3.62	11.26	15.41
	**Mental Health**	6.15	0.72	4.50	6.33
	**Noncommunicable**	28.71	13.05	13.03	31.89
	**Neoplasms**	1.23	0.00	0.36	1.41
**Deceased prior to discharge from unit**	**Trauma**	9.81	33.33	31.25	8.16
	**Infectious**	23.73	0.00	18.75	24.15
	**Mental Health**	4.11	0.00	0.00	4.08
	**Noncommunicable**	58.55	66.67	43.75	59.87
	**Neoplasms**	3.80	0.00	6.25	3.74
**Survived to discharge to unit**	**Trauma**	50.43	83.70	70.29	46.27
	**Infectious**	14.07	3.70	11.11	14.75
	**Mental Health**	6.29	0.74	4.59	6.51
	**Noncommunicable**	26.76	11.86	12.44	29.82
	**Neoplasms**	1.06	0.00	0.24	1.24

Table 3 displays the World Health Organization (WHO) data from 2012 regarding mortality from the overall disease burden in Madagascar. This is a population-level dataset, and therefore its scope is far greater than simply the acute disease burden. Furthermore, it includes pediatric and pregnancy-related disorders that primarily present to the adjacent Centre Mère-Enfant, not investigated during this study.

**Table 3:** Top Causes of Mortality in Madagascar (Source: World Health Organization Global Health Estimates (GHE) 2012).

GHE Code(s)	GHE Cause(s)	% of total deaths(n = 159,800)
148	Injuries	10.1%
2, 38	Infectious and parasitic diseases & Respiratory Infections	36.0%
81	Mental and Behavioral Disorders	0.3%
59	Noncommunicable Diseases	39.4%
60, 78	Neoplasms	8.3%

Table 4 demonstrates a clear increase in the frequency of infectious disease during the rainy season in Madagascar, ranging from November to April. Across all age groups, the frequency of infectious disease rose 3.3% during this time period. In the less than five years of age category the frequency increased by a factor of 2.6, with an overall increase of 3.73%.

**Table 4:** Seasonal Patterns of Traumatic and Infectious Disease.

		All ages	<5 years	<18 years	≥18 years
**Annual**	Trauma	47.94%	82.61%	69.55%	43.65%
	Infectious	14.67%	3.62%	11.26%	15.41%
**Rainy Season (November—April)**	Trauma	45.06%	78.00%	66.31%	41.04%
	Infectious	16.44%	6.00%	13.37%	17.12%
**Dry Season (May—October)**	Trauma	50.42%	85.23%	72.13%	45.94%
	Infectious	13.14%	2.27%	9.57%	13.91%

### Top Diagnoses

Table 5 shows the top 25 CCS-coded diagnoses seen across all age groups at CHUM. Tables 6, 7 and 8 show the corresponding top 25 CCS-coded diagnoses stratified by age group.

**Table 5:** Top 25 Diagnoses: All Age Groups.

CCS Category	Number of Patients	Percentage
Superficial injury; contusion	670	13.04%
Open wounds of head, neck, and trunk	341	6.64%
Open wounds of extremities	297	5.78%
Intracranial injury	241	4.69%
Injury and poisoning (other)	229	4.46%
Somatoform disorders	157	3.06%
Other gram negative septicemia (typhoid fever)	120	2.34%
Intestinal infection	114	2.22%
Alcohol-related disorders	100	1.95%
Poisoning by other medications and drugs	92	1.79%
Appendicitis and other appendiceal conditions	87	1.69%
Hypertension with complications and secondary hypertension	85	1.65%
Pneumonia (except that caused by TB or STD)	76	1.48%
Other infections; including parasitic *	75	1.46%
Heart failure	71	1.38%
Infectious and parasitic diseases (unspecified)	65	1.27%
Tuberculosis	63	1.23%
Open wounds	63	1.23%
Convulsions	55	1.07%
Poisoning by nonmedicinal substances	54	1.05%
Abdominal pain	51	0.99%
Calculus of kidney	48	0.93%
Fracture of radius and ulna	48	0.93%
Intracranial hemorrhage (nontraumatic)	47	0.91%
Fracture of tibia and fibula	46	0.90%

* Malaria comprised 85.3% of the CCS code 1.4, “other infections; including parasitic”.

**Table 6:** Top 25 Diagnoses: < 5 years.

CCS Category	Number of Patients	Percentage
Superficial injury; contusion	37	26.81%
Intracranial injury	18	13.04%
Open wounds of head; neck; and trunk	15	10.87%
Injury and poisoning (other)	12	8.70%
Open wounds of extremities	11	7.97%
Fracture of tibia and fibula	5	3.62%
Inguinal hernia with obstruction or gangrene	3	2.17%
Other fracture of lower limb	3	2.17%
Burns	2	1.45%
Intestinal obstruction without hernia	2	1.45%
Lower gastrointestinal disorders	2	1.45%
Inguinal hernia	2	1.45%
Concussion	2	1.45%
Diseases of the respiratory system	2	1.45%
Anal and rectal conditions	2	1.45%
Somatoform disorders	1	0.72%
Intestinal infection	1	0.72%
Other infections; including parasitic *	1	0.72%
Infectious and parasitic diseases (unspecified)	1	0.72%
Open wounds	1	0.72%
Convulsions	1	0.72%
Fracture of radius and ulna	1	0.72%
Joint disorders and dislocations; trauma-related	1	0.72%
Fracture of upper limb	1	0.72%
Fracture of lower limb	1	0.72%

* Malaria comprised 85.3% of the CCS code 1.4, “other infections; including parasitic”.

**Table 7:** Top 25 Diagnoses: < 18 years.

CCS Category	Number of Patients	Percentage
Superficial injury; contusion	170	20.14%
Intracranial injury	70	8.29%
Open wounds of extremities	62	7.35%
Open wounds of head; neck; and trunk	61	7.23%
Injury and poisoning (other)	47	5.57%
Appendicitis and other appendiceal conditions	32	3.79%
Fracture of radius and ulna	29	3.44%
Somatoform disorders	26	3.08%
Poisoning by other medications and drugs	25	2.96%
Fracture of tibia and fibula	19	2.25%
Poisoning by nonmedicinal substances	19	2.25%
Other gram negative septicemia (typhoid fever)	14	1.66%
Intestinal infection	12	1.42%
Convulsions	10	1.18%
Fracture of lower limb	9	1.07%
Fracture of humerus	9	1.07%
Poisoning	9	1.07%
Open wounds	8	0.95%
Fracture of upper limb	8	0.95%
Inguinal hernia	7	0.83%
Other infections; including parasitic *	7	0.83%
Infectious and parasitic diseases (unspecified)	7	0.83%
Lower gastrointestinal disorders	6	0.71%
Abdominal pain	6	0.71%
Inguinal hernia with obstruction or gangrene	5	0.59%

* Malaria comprised 85.3% of the CCS code 1.4, “other infections; including parasitic”.

**Table 8:** Top 25 Diagnoses: ≥ 18 years.

CCS Category	Number of Patients	Percentage
Superficial injury; contusion	498	11.68%
Open wounds of head; neck; and trunk	279	6.54%
Open wounds of extremities	233	5.47%
Injury and poisoning (other)	179	4.20%
Intracranial injury	169	3.96%
Somatoform disorders	131	3.07%
Other gram negative septicemia (typhoid fever)	106	2.49%
Intestinal infection	102	2.39%
Alcohol-related disorders	90	2.11%
Hypertension with complications and secondary hypertension	85	1.99%
Pneumonia (except that caused by TB or STD)	73	1.71%
Heart failure	69	1.62%
Other infections; including parasitic *	67	1.57%
Poisoning by other medications and drugs	66	1.55%
Tuberculosis	63	1.48%
Infectious and parasitic diseases (unspecified)	58	1.36%
Appendicitis and other appendiceal conditions	55	1.29%
Open wounds	55	1.29%
Intracranial hemorrhage (nontraumatic)	47	1.10%
Abdominal pain	45	1.06%
Convulsions	44	1.03%
Calculus of kidney	44	1.03%
Gastrointestinal hemorrhage	43	1.01%
Occlusion of cerebral arteries	41	0.96%
Acute cerebrovascular disease	40	0.94%

* Malaria comprised 85.3% of the CCS code 1.4, “other infections; including parasitic”.

### Trauma

Overall, traumatic injury and poisoning accounted for the greatest number of ED visits across all age categories. Superficial injuries were the most common injury types. Abrasions (35%), contusions (59%), and hematomas (5.7%) together accounted for 99.9% of all superficial injuries. Intracranial injuries were also very common, accounting for 4.7% of visits overall.

### Infectious

Infectious disease accounted for 14.7% of all patient visits. Typhoid fever, classified as CCS code 1.1.2.4, “Other gram negative septicemia”, was the most common infectious disease, accounting for 16% of all infections. Second most common was intestinal infection, which accounted for 15.1% of all infections.

Infectious disease was the most lethal pathology in patients above 18 years of age, causing 24.2% of all deaths in this age group. No patients under 5 years of age died as a result of an infectious cause in this study.

### Mental Health

The majority of psychiatric disorders were classified as CCS category 5.15.7, “Somatoform Disorders”. Ninety-four percent of the diagnoses classified under this category were recorded in the registry in French as “dystonie neurovegetative”. This is an archaic psychiatric term with no exact equivalent in the current Diagnostic and Statistical Manual of Mental Disorders (DSM), but is commonly used in Madagascar for a constellation of symptoms including somatization and anxiety.[13]

Table 9 shows patient general disposition trends. The admission rate at CHUM, often regarded as a rough measure of acuity, is 43%.

**Table 9:** Patient Disposition—General.

Disposition	N	Percentage
Discharge	2515	48.9%
Admission	2208	43.0%
Death	316	6.15%
Left prior to discharge	68	1.32%
Transfer to outside facility	31	0.60%

Table 10 shows the receiving services of admitted patients. Trauma patients accounted for the highest percentage of all admissions, at 19.1%. This was followed closely by internal medicine (including infectious disease), which received 16.1% of admissions.

**Table 10:** Patient Disposition—Admission Services.

Admission Service	N	Percentage
Trauma	422	19.1%
Internal Medicine	355	16.1%
Surgery	280	12.7%
Cardiology	244	11.1%
Neurology / Psychiatry	183	8.30%
Gastroenterology	149	6.76%
Pulmonology	143	6.49%
Gynecology	155	7.03%
Otolaryngology	124	5.62%
Urology	84	3.81%
Obstetrics (Labor and Delivery)	40	1.81%
Pediatrics	25	1.13%
Dermatology	1	0.05%

### Mortality

The deaths listed in the registry include those that occurred at any point during the patient's stay in the Servics des Urgences et Soins Intensifs, which is a combined emergency department and intensive care unit. The mean time of death was 17:45 (95% CI ± 0:50), with a standard deviation of 6:55. In order to make a more meaningful comparison between mortality at the CHUM and other emergency care centers, we have used Table 11 to delineate whether death occurred within or after the first 24 hours. One hundred eighty-one deaths out of the total 316 occurred within the first 24 hours, and are listed in Table 11 as “Early Death (within 24hrs)”. One hundred thirty-four deaths occurred after the first 24 hours, but prior to discharge from unit, and are listed as “Delayed Death (> 24 hours)”. One patient had an unknown date and time of death. Data on mortality after discharge from the unit was not gathered.

**Table 11:** Timing of Death.

Timing of Death	N	Percentage
Early Death (within 24 hours)	181	57.3%
Delayed Death (> 24 hours)	134	42.4%
Unknown time of death	1	0.3%

Mean time of death 17:45 (95% CI ± 0:50), standard deviation 6:55.

## Limitations

The lack of gender information on children under 18 limits demographic characterization. This is particularly true for children under five years of age, a group that normally presents to a separate pediatric wing of the hospital.

Our use of an international standard classification system facilitates comparison with existing literature; however, limitations of our methodology include capture of only single primary diagnoses, limiting the ability to characterize prevalence of conditions. For example, HIV patients may be listed only by presenting syndrome or a specific secondary infection and would not be identified as having HIV. Similarly, many trauma patients would have only the primary injury documented. Further research capturing multiple diagnoses is needed to better characterize the burden of acute disease.

Finally, this was a retrospective chart review and is therefore subject to bias surrounding the use of such data, namely an inability to establish causal relationships secondary to unknown confounding variables. Accordingly, we do not attempt to establish any such relationships, this is a purely descriptive study.

## Discussion

We found a slight male predominance in our study at 53.5%; other regional and global studies have reported a higher male prevalence.[14] Sixteen percent of patients in our study population were under 18, and 2.7% were under 5; this is lower than similar regional studies.[7]

Similar to burden of disease studies performed in Kenya and Tanzania, traumatic disease was the most common acute pathology overall, followed by infectious etiologies.[7,15] However, the age-specific distribution of diagnoses was reversed from that found in Tanzania, where trauma was more common in adults and infectious disease in children.

The admission rate at the CHUM was 43%, which is 3.3 times higher than the United States.[16] This trend of high admission rates is commonly observed in low- to middle-income countries. A study from a Tanzanian teaching hospital, similar in size and relative geographical location, showed a similar rate of 46%, but admission rates as high as 62.6% have been reported in Uganda and 52.9% Kenya.[6,17,18] Analyzing these regional similarities and differences is currently difficult, as there is a paucity of literature containing precise codes for diseases. This highlights the need for reporting based on a standardized system, such as the ICD-9-based CCS structure implemented here.

Pediatric patients do not present to the CHUM’s Unité des Urgences et Soins Intensifs as there is a separate acute care intake area for this demographic. Accordingly, pediatric data from this study cannot be generalized to the entirety of the CHUM’s catchment area. For example, there were no deaths in children under five from infectious disease in our study. This is in contrast to WHO regional data in which infections are one of the leading causes of mortality in very young children.[8]

The range of infectious diseases in Madagascar is distinct from other regional studies. Malaria accounted for less than 10% of all infections, and HIV/AIDS was never listed as the primary diagnosis. This may be due to a combination of negative social stigma creating resistance to listing the diagnosis, to the use of only single primary diagnoses as mentioned above, or to the relatively low national prevalence (1.7% amongst ages 15 to 49) [19]. Mental health was one the third most common general category of disease presentations (after breaking down noncommunicable disease into more granular pathologies). This result is similar to findings from Muhimbili National Hospital in Dar Es Salaam, Tanzania, where mental illness was the most common overall disease pathology[7]. The lack of alternate outpatient care and resources for these patients may explain the high prevalence of mental illness seen in the Emergency Department.

When comparing mortality rates between our population and the existing WHO global burden of disease dataset (Tables 2 and 3, respectively), it is important to emphasize that our data represents a facility-based utilization review, unlike the WHO population-based data. Our goal is to characterize specifically the burden of *acute* disease addressed by the emergency care system in Madagascar. We offer this comparison only for perspective in examining the burden of acute disease with respect to the overall disease burden. Traumatic injury accounted for similar mortality rates between the WHO population-level (10.1%) and our facility-level (9.8%) data. Infectious disease mortality represented a relatively lower fraction at the CHUM (23.7%) than at the population-level (36%). Mortality from mental health disorders, largely comprised of suicide by pesticide ingestion, accounted for 4.1% of our deaths, compared to 0.3% in the overall population. Finally, we observe that our mortality from noncommunicable diseases (NCDs) was higher (58.6%) than in the WHO-reported overall disease burden (39.4%). These disparities are predominantly the result of the inherent differences in architecture between facility-based reviews and population-level data collection. They highlight the importance of performing such a review in order to define the disease burden capable of being targeted by an emergency care system.

## Conclusion

This study describes the burden of acute disease at a large referral center in northern Madagascar. The Centre Hospitalier Universitaire de Mahajanga sees a high volume of acutely ill and injured patients. Similar to other reports from the region, trauma is the most common pathology observed, though infectious disease was responsible for the majority of adult mortality. Typhoid fever other intestinal infections were the most lethal CCS-coded pathologies. By utilizing a widely understood classification system, we are able to highlight contrasts between Mahajanga’s acute and overall disease burden as well as make comparisons between this region and the rest of the globe. We hope this study will serve to guide the development of context-appropriate emergency medicine systems in the region.
